# Genomic insight into the influence of selection, crossbreeding, and geography on population structure in poultry

**DOI:** 10.1186/s12711-022-00775-x

**Published:** 2023-01-20

**Authors:** Zhou Wu, Mirte Bosse, Christina M. Rochus, Martien A. M. Groenen, Richard P. M. A. Crooijmans

**Affiliations:** 1 Animal Breeding and Genomics, Wageningen University and Research, Wageningen, The Netherlands; 2 Present Address: The Roslin Institute and Royal (Dick) School of Veterinary Studies R(D)SVS, University of Edinburgh, Easter Bush, Midlothian, EH25 9RG UK; 3 Present Address: Centre for Genetic Improvement of Livestock, Animal Biosciences, University of Guelph, Guelph, ON Canada

## Abstract

**Background:**

In poultry, the population structure of local breeds is usually complex mainly due to unrecorded breeding. Local chicken breeds offer an interesting proxy to understand the complexity of population structure in the context of human-mediated development of diverse morphologies and varieties. We studied 37 traditional Dutch chicken breeds to investigate population structure and the corresponding genomic impact using whole-genome sequence data.

**Results:**

Looking at the genetic differences between breeds, the Dutch chicken breeds demonstrated a complex and admixed subdivided structure. The dissection of this complexity highlighted the influence of selection adhering to management purposes, as well as the role of geographic distance within subdivided breed clusters. Identification of signatures of genetic differentiation revealed genomic regions that are associated with diversifying phenotypic selection between breeds, including dwarf size (bantam) and feather color. In addition, with a case study of a recently developed bantam breed developed by crossbreeding, we provide a genomic perspective on the effect of crossbreeding.

**Conclusions:**

This study demonstrates the complex population structure of local traditional Dutch chicken, and provides insight into the genomic basis and the factors involved in the formation of this complexity.

**Supplementary Information:**

The online version contains supplementary material available at 10.1186/s12711-022-00775-x.

## Background

Following the process of domestication, chicken populations have been diversified with distinct morphological features during subsequent worldwide agriculture revolutions. During this process, chickens have been selectively bred for specific types of management, which led to genetic differentiation and population subdivision. Based on human interventions, chicken populations can be generally classified into commercial breeds and local traditional breeds. The commercial breeds have been bred under extensive and specialized selection for either egg-laying (layers) or meat production (broilers) [1]. Breeding for local traditional breeds is contingent on diverse and complex mechanisms which lack strict supervision, such as migration, random drift, geographic dispersal followed by environmental adaptation, and selective breeding adhering to breed characteristics [2–5]. For instance, several studies have shown that geographic dispersal distances can lead to genetic differentiation in local indigenous populations [6, 7], as well as in pure breeds [8]. The most extreme influence of geographic pattern can lead to isolation and speciation which is known as allopatry [9, 10].

Various factors or forces that influence population structure may coincide with each other and/or outcompete one another, which is subsequently comprised in the complexity of population structure. To understand the complex structure of populations, many studies have been performed to untangle the differentiation between populations by initially looking for genomic regions with divergent genetic signals. Identification of genetic differentiation serves as a good starting point to understand the genetic basis that underlies phenotypic variation. Phenotypes such as body stature, comb type, and coat/plumage pattern may reflect the history of selection [11–15]. Genomic analytical approaches, such as detection of signatures of selection by identifying genomic regions with pronounced genetic differentiation between populations or breeds, enable us to understand the differentiation and complex structure in a population [16].

In the Netherlands, historic chicken resources have been admired and received attention from the sixteenth century onwards, and continued to be managed in diverse forms [4, 17]. The long tradition of breed development resulted in various organizations (private and governmental) making efforts to use and preserve native breeds as well as their genetic resources [4]. Dutch chicken breeds are defined mostly based on geographic location (e.g., Groningen Mew Fowl) and phenotypic characteristics (e.g., Dutch Polish Bearded). These breeds are subdivided by management type and historical clustering, which was profiled in many relevant studies [3, 14, 17, 18]. This historical clustering includes past-productive breeds that have been developed for rapid production (especially egg production and dual purpose), ornamental breeds used for fancy breeding, and country fowls represented by primitive traditional breeds that have limited management history. At the same time, Dutch chicken breeds include a unique variety of bantam forms (a dwarf phenotype with 50–60% reduced body weight). Besides the *traditional* or *true* bantam breeds, Dutch chicken breeds exhibit an important history of bantam crossbreeding, so-called bantamization. The bantamization process used donors from existing bantams and/or neo-bantams of local breeds to create corresponding neo-bantams, the dwarf counterparts of native ‘large’ chickens [3, 11, 19]. As a common practice, bantam crossbreeding was followed by repeated backcrossing and selection, in order to maintain similar characteristics between neo-bantams and ‘large’ chickens, which has proven to effectively dilute the contribution from bantam ancestries [3, 11]. From a genomic perspective, because of backcrossing, the introgressed genomic segments are expected, over time, to be broken by recombination. Therefore, the genomic composition of neo-bantams is expected to contain only a few genomic segments derived from the original bantam donors, apart from loci that contribute to the bantam phenotype.

In this study, we focused on multiple factors that may play a significant role in recent breed formation revealing the complex population structure of Dutch traditional chicken breeds. We used whole-genome sequence data, representing the genetic basis of various breeds to investigate the population structure between breeds, and the effect of management type, geographic distribution, and phenotypic selection. Lastly, we demonstrate the recent hybrid history and provide a genomic perspective on the effect of selective breeding by using the case of a neo-bantam breed (Drenthe Fowl bantam).

## Methods

### Sampling of chickens

The chickens sampled for whole-genome sequencing included 136 individuals from 37 breeds, in which the neo-bantam and their normal-sized counterparts were considered as different breeds (see Additional file 1: Table S1). Historical breed information of Dutch chicken breeds has been described in previous studies, including breed history [17–19], estimation of genomic diversity [3], bantamization history, and body weight [11]. Breeds within the same cluster have similar management or breeding goals. The historical classification of Dutch breeds has been reported in Elferink et al. [18] and Bortoluzzi et al. [3]: CL1 past-productive, CL2 ornamental, CL3 country fowl, and CL4 Lakenvelder and Lakenvelder bantam. For almost every Dutch traditional breed, there is a corresponding bantam counterpart (neo-bantam) included in the cluster, with the exception of the Chaam Fowl. The two true bantam breeds, the Dutch Bantam and Eikenburger bantam, are assigned to the cluster of country fowl.

### Whole-genome sequencing and processing of genetic variants

Whole-genome paired-end sequencing (PE125) was performed on the Illumina HiSeq 3,000 platform, with insert sizes of 350 bp. The processing of whole-genome sequence data was conducted following an in-house analysis pipeline. In brief, raw sequencing reads were trimmed by Sickle [20] then mapped to the chicken reference assembly, build GRCg6a (GenBank Accession: GCA_000002315.5) using BWA-MEM (V0.7.17) [21]. Duplicated reads were marked and removed using sambamba V0.6.3 [22]. Whole-genome single nucleotide polymorphisms (SNPs) and insertions and deletions (InDels) were genotyped using the Freebayes software [23]. After filtering for base quality and genotype quality, we further filtered the variants using the following criteria: a minor allele frequency (MAF) higher than 1% and a call-rate higher than 80%.

### Haplotype sharing (identity-by-descent) detection

We used the genotype phasing program Beagle (version 5.0) [24] to construct haplotypes for all individuals with a sliding window size of 0.02 cM and a 0.01 cM overlap between adjacent windows with 12 iterations. The chromosomal genetic distances were based on Elferink et al. [25]. However, this genetic map does not cover all the chromosomes. Therefore, only the chromosomes with a reported genetic map were included in this analysis, and uninformative chromosomes (*Gallus gallus* chromosomes GGA16 and GGA30-33), linkage groups without chromosomal location, and the sex chromosomes (Z and W) were excluded. An exception is the genetic map for GGA25 which was derived from an earlier study by Groenen et al. [26] because it was missing in the study of Elferink et al. [25].

Detection of identity-by-descent segments was based on the haplotype of individuals using the software package Refined-ibd [27]. The aim of this analysis was to detect identity-by-descent segments derived from the same population rather than from a single common ancestor. The identity-by-descent segments were detected using the following requirements: window size of 0.06 cM, length = 0.03 cM, trim for length < 0.001 cM, and LOD score > 3. To infer the relative fraction of haplotype sharing in regions across the genome, we computed the relative identity-by-descent (rIBD) frequency following the methods from Bosse et al. [28]. The rIBD in this study was calculated between the DrFwB and the two donors (bantam donor, DB; and normal-sized donor, DrFw) in bins of 10 kb. The rIBD in different bins of the genome was compared and plotted by chromosome (see below for more details).

### Population structure analysis

Population structure analyses were performed using three approaches: a principal component analysis (PCA), a population phylogeny constructed based on Reynold’s distance, and an admixture analysis: (1) the PCA was computed using the PLINK (V1.9) software [29] with autosomal variants, and visualized using the R package “plotly” [30]; (2) the pairwise Reynolds’ genetic distances between breeds were computed as in Fariello et al. [31] and the phylogenetic tree was fitted using the neighbor-joining algorithm; we visualized genetic distances by using FigTree V1.4.4 (http://tree.bio.ed.ac.uk/software/figtree/); the phylogenetic relationship was used in the FLK and hapFLK analysis; and (3) we used the genetic analysis software ADMIXTURE to estimate ancestry in individuals [32], the software was run with different numbers of ancestral populations, and representative outputs of K = 4 and K = 6 are presented in this paper.

### Isolation-by-distance test

**Fig. 1:**
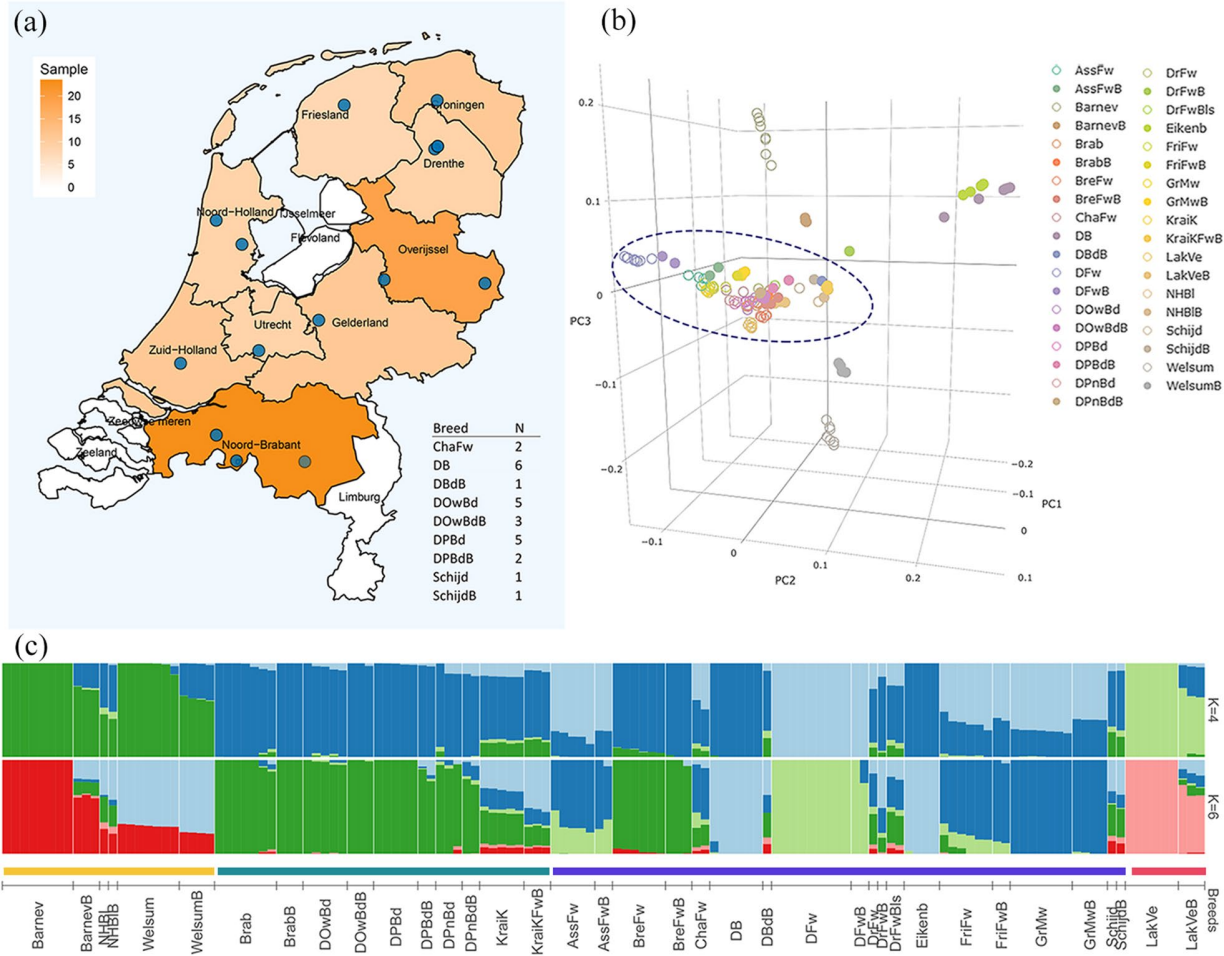
Population structure in Dutch chicken breeds. **a** The map shows the provinces in the Netherlands from where the breeds originated. The blue circles on the map represent the known region of origin of the samples, whereas those breeds without any clear geographic information are listed in table format at the bottom-right. **b** Principal component analysis showing the first three principal components. **c** Admixture analysis with K = 4 and 6. The ancestry coefficients of each individual are shown by vertical bars. The horizontal bars display the different clusters, CL1 (yellow), CL 2 (green), CL3 (purple), and CL4 (Lakenvelder and its bantam) (red). Abbreviated breed names are in Additional file 1: Table S1

The geographical distance is determined by haversine distance given the longitude and latitude of the location of the origin of the breeds using the “geosphere” package [33] in R V3.6.1. The individual pairwise genetic distance was computed as 1 minus identity-by-state values (1-ibs), using the autosomal whole-genome variants with the command (-distance square 1-ibs) in PLINK (V1.9). Because of the unknown geographic location for nine of the 37 breeds, these were excluded from the isolation-by-distance test. These nine breeds are listed in table format in Fig. 1a. We performed a mantel test and determined the level of significance with permutation for 9999 times. We tested the isolation-by-distance with individuals from all populations, and examined it within groups based on the three historical clusterings, including past-productive, ornamental, and country fowl. Because CL4 only contained Lakenvelder and its bantam, we excluded it from the isolation-by-distance test. To show the haplotype similarity within clusters and between clusters, the detection of identity-by-descent segments was performed in each cluster accordingly. The count and the length of identity-by-descent segments between individuals were visualized for comparisons within the cluster and between this cluster and the rest.

### FLK and hapFLK

The FLK and hapFLK analyses were conducted to detect the signatures of selection between different populations [31, 34]. The autosomal variants were pre-processed before computing FLK and hapFLK. To save computational time, we filtered out the variants with a MAF lower than 8% and performed linkage equilibrium pruning (r^2^ > 0.3) using PLINK software (-indep-pairwise 10 10 0.3). In total, we ended up with 2.30 million variants covering the genome. The hierarchical structure (Reynolds’ distance matrix) of the populations was accounted for in the genomic scans for signatures of selection. For the single-marker analysis, we performed the FLK test on all variants by chromosome. As for the haplotype-based FLK (hapFLK) test, we set the number of haplotype groups in the population to K = 15, as suggested by Boitard et al. [16], where the P-values were derived by scaling the hapFLK statistic to a chi-square distribution as described in their paper. The significant signals were determined at a false discovery rate (FDR) of 5%.

We annotated the variants that were within 5-kb up- or downstream regions from a gene or regulatory element (Ensembl gene sets version 95). Selective sweeps that were annotated with more than one significant variant were considered for further investigation. First, we compared the genes with the Online Mendelian Inheritance in Animals (OMIA) dataset, searching for “likely known” causative genes for Mendelian traits in chicken (https://omia.org/home/). Second, a gene ontology enrichment analysis was carried out using PANTHER v.11 [35] based on the chicken to human one-to-one orthologues. Gene enrichment analysis was then performed by using the package clusterProfiler [36].

### Haplotype sharing in the case study

In the case study of Drenthe Fowl bantam chicken, haplotype sharing was measured by identity-by-descent among the three breeds (Dutch Bantam, Drenthe Fowl, and Drenthe fowl bantam). The pairwise identity-by-descent segments between two breeds were estimated by bins of 10 kb and then normalized by the possible count between these two breeds (nIBD). The rIBD was computed by comparing the nIBD between DB and DrFwB (nIBD_DB_DrFwB_) with the nIBD between DrFw and DrFwB (nIBD_DrFw_DrFwB_). A positive rIBD shows more haplotype sharing between DB and DrFwB than between DrFw and DrFwB, whereas a negative rIBD shows more haplotype sharing between the counterparts (i.e., DrFw and DrFwB) correspondingly. In order to compare the rIBD pattern in another neo-bantam breed with a similar crossbreeding history, we selected individuals from the GrMwB and GrMw breeds to show the introgression signals. To keep the samples comparable, we chose one individual from each breed, and the average coverage of the samples should be adequate and as good as the coverage of DrFwB. Likewise, we computed the rIBD between DB, GrMwB, and GrMw. The DB introgressed regions were highlighted in the visualization.

## Results

### Complex and admixed population structure

In total, we analyzed whole-genome sequence data of 136 chickens from 37 breeds and assessed population structure (Fig. 1). Based on previous studies, Dutch traditional chicken breeds can be grouped into three historical clusters according to the historical and conventional management classification. The cluster of past-productive fowl (CL1) is represented by 24 individuals from six breeds, the ornamental breeds (CL2) comprise 38 samples from 10 breeds, and the country fowl cluster (CL3) is represented by 65 chickens from 19 breeds (see Additional file 1: Table S1). Besides these clusters, nine individuals from the Lakenvelder breed and its bantam counterpart were included in the analyses as well (CL4) due to a unique history and genetic composition relative to others [3]. We used three approaches to assess whether the genetic population structure matches the historical clustering: a principal component analysis (PCA) decomposing genomic variants, a neighbor-joining tree (NJ-tree) illustrating the Reynold’s distance between breeds, and an admixture analysis estimating the proportion of ancestry.

The PCA revealed that the PC1 (12.63% of variance explained) separated three breeds and their bantam counterparts from the other breeds: WelSummer (WelSum), Barnevelder (Barnev), and North Hollands Blue (NHBl) (Fig. 1b). These three breeds are considered as past-productive breeds in the Netherlands, therefore confirming this historical clustering. The PC2 (7.79% of variance explained) separated the two true bantam breeds, Dutch bantam (DB) and Eikenburger bantam (Eikenb), from the other breeds. The percentage of variance explained by the PC3 was 7.35%, but the remaining breeds, including ornamental breeds and country fowl, clustered closely together in the PCA plot, suggesting an underlying genetic relationship. The unique population structure is supported by the unrooted NJ-tree (see Additional file 2: Fig. S1). The historical clustering of the breeds was also demonstrated in the population phylogeny analysis, with the branch length of each breed showing the genetic changes since the branch point diversification between them.

The ADMIXTURE analysis complements the other two approaches. The results of ADMIXTURE (Fig. 1c) subdivided the clusters, showing varied proportions of presumed ancestry. In agreement with the PCA and phylogenetic analyses, the clustering of past-productive breeds (CL1) and the group of Lakenvelder (CL4) are clearly separated at both K = 4 and K = 6, whereas the ornamental breeds (CL2) and country fowl (CL3) seemed to be more related. Strong admixture signals for breeds such as Twentse Fowl (KraiK), Schijndelaar (Schijd) and Drenthe Fowl (DrFw), as well as their bantams, showed population subdivision at K = 6, implying their complex ancestry. Compared with other breeds, the country fowls showed varying levels of admixture. Among them, the Dutch Bantam and Eikenburger were separated from the rest, which agrees with PC2 in the PCA. In addition, the Kraienkoppe Fowl (BreFw) and its bantam, belonging to country fowl, shared a large proportion of genetic ancestry with the ornamental chickens (CL2).

### Isolation-by-distance

To study the role of geographic dispersal in the population structure and the genetic differences between Dutch breeds, we performed an isolation-by-distance test to assess the correlation between genetic and geographic distances. The isolation-by-distance test was performed for the dataset with all the Dutch breeds, as well as within only the three historical clusters of breeds.

**Fig. 2:**
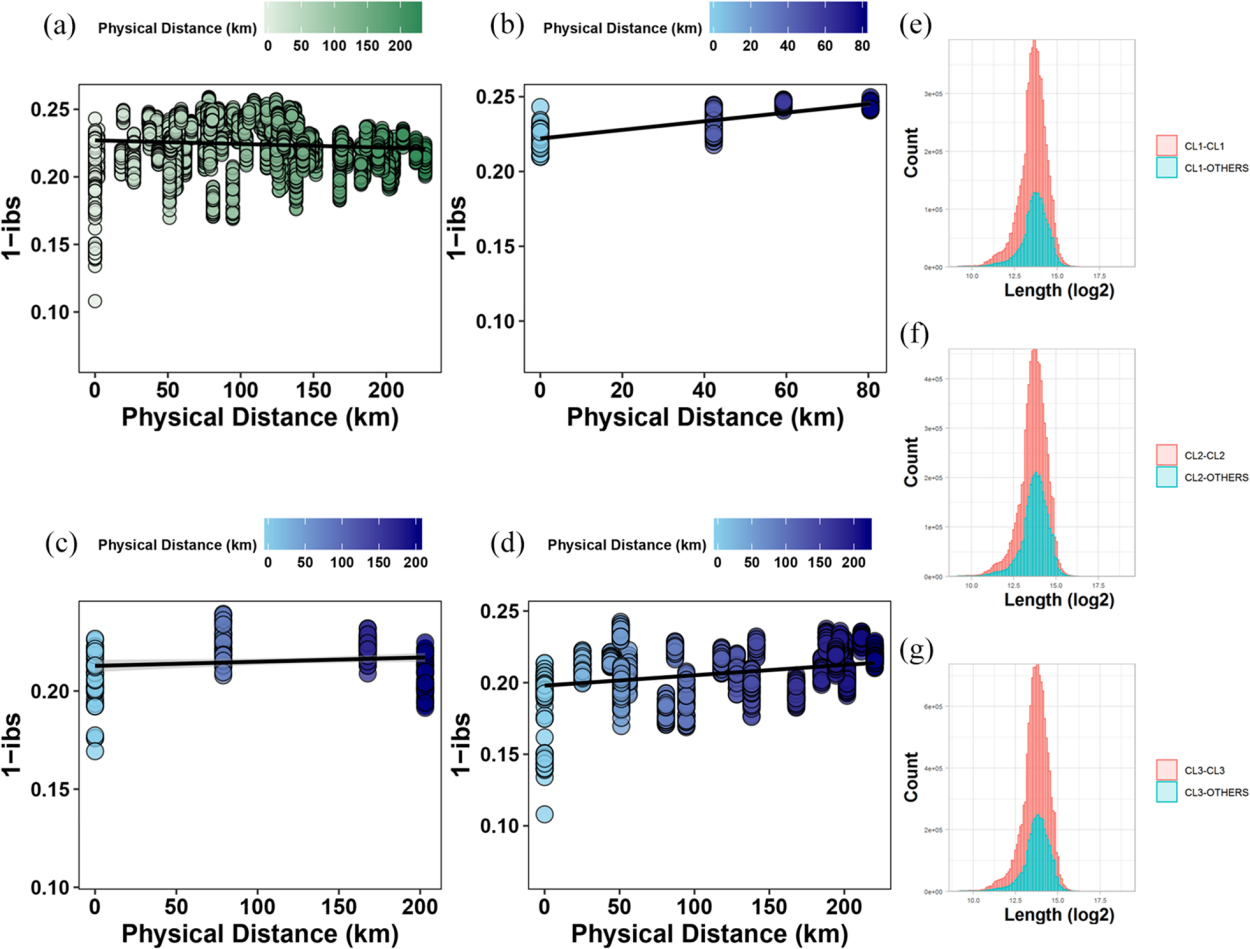
Correlation between genetic distance (1-ibs) and geographic distances (in km): **a** between all breeds from the Dutch population and (**b**–**d**) between breeds in CL1, 2 and 3, and (**e**–**g**) distribution of shared identity-by-descent blocks identified within each subdivided cluster (red) and between clusters (green)

The result of the isolation-by-distance test among all Dutch chicken breeds showed a nonsignificant pattern of isolation-by-distance (mantel r = − 0.09, P = 0.99), suggesting no clear correlation between genetic and geographical distance across the population (Fig. 2a). Analysis of the isolation-by-distance pattern within historical clusters revealed a strong positive correlation in CL1 (mantel r = 0.73, P = 1 × 10^–4^) and CL3 (mantel r = 0.33, P = 1 × 10^–4^), while breeds in CL2 showed a positive but rather weak correlation (mantel r = 0.13, P = 0.034) (Fig. 2b–d). The significant correlation between the genetic and geographical distances within clusters suggested that breeds located at increasing geographical distance also display increased genetic differences. This suggests that geographic distance plays a role in the genetic difference within clusters. Whereas across clusters, the different management purposes can impose a genetic “barrier”, limiting the genetic exchange between clusters. To test our hypothesis of a management-based “barrier”, we examined haplotype sharing (identity-by-descent) across breeds within and between clusters. Haplotype sharing of identity-by-descent fragments has been used to reveal recent relatedness and demographic history between individuals in previous studies [8, 28]. Within the three clusters, the haplotype sharing patterns were consistent. We found extensively shared haplotypes between individuals within clusters rather than between individuals belonging to different clusters (Fig. 2e–g).

### Detection of FLK and hapFLK signals

One of the causes of the complex population structure of Dutch traditional chicken breeds is selective breeding adhering to desired traits and/or management types. The breed features shared between multiple breeds have led to extensive crossbreeding and recycling of the genetic materials of these phenotypes. Over the past decades and among the many desired phenotypic features, selection has focused on downsizing the local breeds and increasing morphological varieties, especially color varieties. To identify genomic regions that underlie the desired features, we tested the dataset of 37 breeds for genetic differentiation of all kinds of breed-specific or breed-overlapping traits. We applied the FLK approach (extended LK test) using both the single variant and haplotype information. The single variant approach FLK was used to detect genomic regions that display genetic differentiation between populations, such as signatures of selection, while accounting for the hierarchical structure of the populations [34]. Likewise, the haplotype-based approach, hapFLK, was used to detect differences in haplotype frequencies.

**Fig. 3:**
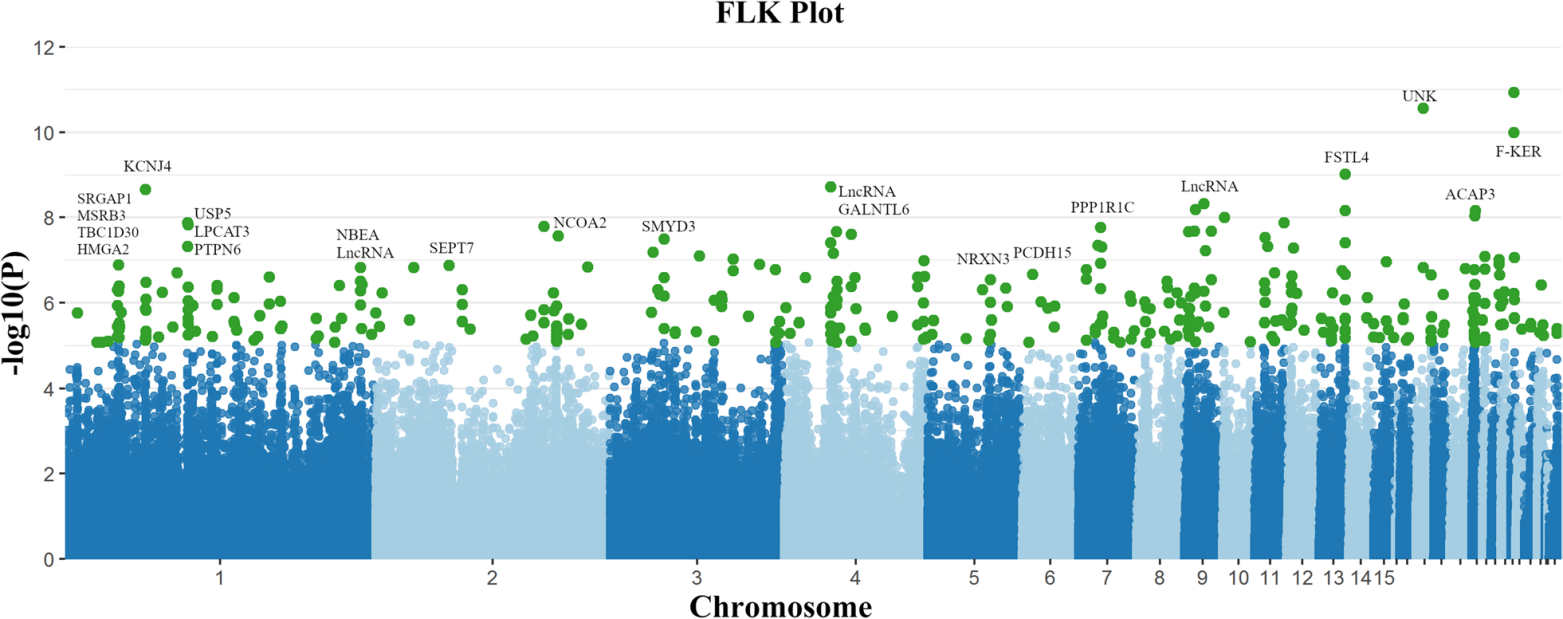
Manhattan plot of FLK across the genome detected in Dutch chicken breeds. Significant variants (FDR > 5%) of FLK are indicated with green dots

We observed 387 significant signals (FDR 5%) in 299 genes across the genome by using the FLK test, suggesting potential genetic differentiation in the population (Fig. 3). Among the genes identified by the FLK test, we did not find any GO term that was significantly overrepresented, which can be explained by these breeds being selected for multiple phenotypes rather than one single trait. Compared with previously reported candidate genes associated with the bantam phenotype [11], eight bantam candidate genes overlapped with selective sweep signals of FLK. These signals confirmed that the bantam-related genomic regions were part of a strong selection regime. Interestingly, we found selective sweeps that overlapped with the bantam candidate genes encoding *high mobility group AT-hook 2* (*HMGA2*) and *PR/SET domain 16* (*PRDM16*). These genes have been reported to be associated with body growth and reduced stature (dwarf) in diverse species [37–41], which further supports the strong bantam associated signals in our previous GWAS [11]. Although we did not find hapFLK signals reaching the significance threshold (see Additional file 2: Fig. S2), interesting suggestive signals were observed which supplement the signals discovered by the FLK analysis. The hapFLK revealed signals surrounding the *ENSGALG00000052273* and *ENSGALG00000049778* genes on chromosome 4, encoding two long non-coding RNAs (lncRNAs). In addition, a strong selective sweep was observed around 170.72 Mb on chromosome 1, proximal to the quantitative trait locus (QTL) that is associated with growth and body weight (170.52–172.04 Mb) [42]. Given that the bantam phenotype is one of the most prominent features in the Dutch population, the identified genomic regions associated with body growth and size variation are of great interest. This QTL region is composed of the genes *potassium channel regulator* (*KCNRG*) and *tripartite motif containing 13* (*TRIM13*) and of the microRNAs, *gga-mir-15a* and *gga-mir-16-1* (see Additional file 2: Fig. S2B). Focusing on chromosome 1, we integrated the results of FLK with hapFLK and observed two suggestive signatures of selection surrounding the *SRY-box 10* (*SOX10*), and *SRY-box 5* (*SOX5*) genes.

### Impact of recent crossbreeding: a case study in Drenthe Fowl Bantam

We have addressed the signatures of selection that are related to the introduction of the desired phenotypes and especially the strong selection at bantam associated loci. Therefore, we took a neo-bantam breed as a case study to show the hybrid nature of its genome and the consequence of crossbreeding to introduce desired traits. The bantam-oriented crossbreeding to introduce this dwarf phenotype is an excellent model to understand and predict the introgression of bantam.

Drenthe Fowl bantam (DrFwB) as the corresponding neo-bantam counterpart of the traditional breed Drenthe Fowl (DrFw), has a recent history of the bantam crossbreeding process. According to the bantamization record, DrFwB was created by mating DrFw with existing bantam breeds, particularly the Dutch Bantam (DB) [19]. Thus, the relatively recent crossbreeding for DrFwB represents an ongoing trajectory, resulting in a unique genomic characteristic of the breed. Unlike the other neo-bantam breeds (e.g., Groningen Mew Bantam; GrMwB), which share a similar crossbreeding history with the same presumed donors, DrFwB showed a closer genetic relationship with the bantam donor DB (0.199), while it is more distantly related to its normal-sized counterpart DrFw (0.213). This unique relationship was supported by the admixture signal of DrFwB (Fig. 1c). Because the crossbreeding to create DrFwB is relatively recent, subsequent backcrossing has not yet been able to effectively dilute the component of its bantam donor. The removal of these genomic segments requires time to effectively take place by backcrossing or selection, which enables us to monitor the effect of this ongoing process.

To understand crossbreeding in the context of bantamization, we used the genetic haplotype sharing (identity-by-descent) method to address the contribution of bantam ancestry towards the neo-bantam breed, which is from DB to DrFwB. The number and length of IBD fragments between DB and DrFwB showed abundant segments of shared haplotypes (see Additional file 2: Fig. S3). In contrast, a smaller number of haplotype blocks was shared between the counterparts, DrFw and DrFwB.

**Fig. 4:**
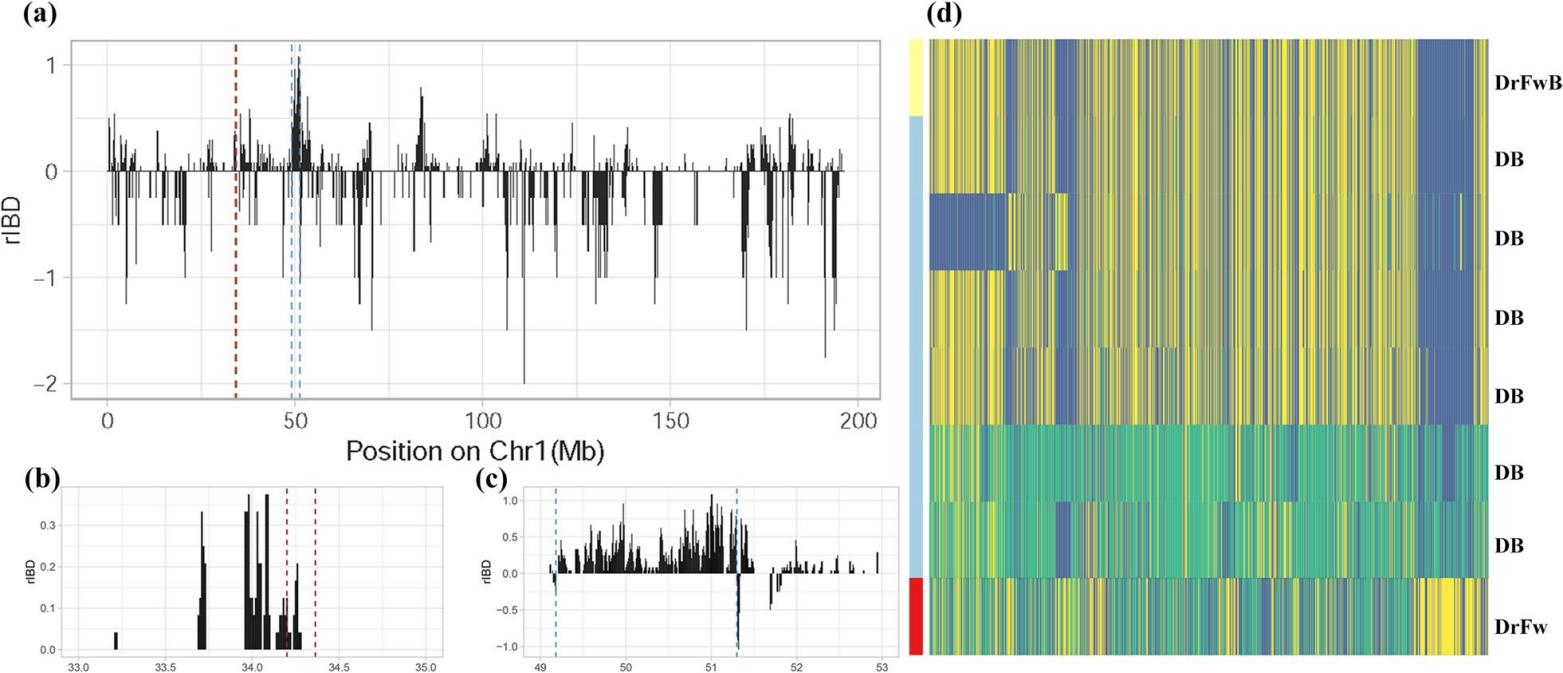
Relative haplotype sharing and genotypes of individuals from DrFwB, DrFw, and DB. **a** Distribution of relative identity-by-descent (rIBD) on chromosome 1, showing the relative fraction of haplotype blocks shared between DrFwB and DB (positive rIBD) and DrFwB and DrFw (negative rIBD). The red and blue dashed lines denote the *HMGA2* related region and a long stretch of DB introgressed region. **b** Zooming in on the *HMGA2* related interval on chromosome 1 highlighted by the red dashed lines. **c** The DB introgressed region on chromosome 1 (49.2–51.3 Mb) is indicated by the blue dashed lines. **d** Individual genotypes of the representative DB introgressed region (50.7–51.0 Mb) are visualized for each locus: homozygous reference allele (blue), homozygous alternative allele (yellow), heterozygous locus (green)

Regional haplotype sharing was summarized by rIBD after normalization to further detect the haplotype sharing signals across the genome (Fig. 4). In general, almost all chromosomes contained regions similar to either the counterpart (DrFw) or the true bantam (DB) donors, which suggests the presence of overall signals of haplotype sharing across the genome (see Additional file 2: Fig. S4). However, when focusing on specific chromosomes (e.g., chromosome 1), we observed large and consecutive stretches of rIBD in DrFwB displaying a higher similarity with DB. To confirm the relative haplotype sharing signals captured by rIBD, we examined the region around the *HMGA2* gene that was previously identified to be underlying the bantam phenotype [11]. As expected, the haplotype blocks around the *HMGA2* gene were almost completely shared between DB and DrFwB, confirming that the bantam-related signals are likely introgressed from bantam donors or the co-ancestral population.

Interestingly, we noted a large region containing extensive and consecutive DB haplotypes between 49.2 and 51.3 Mb on chromosome 1 which outcompeted the bantam associated locus (around the *HMGA2* gene) in terms of length and sequence similarity. The genes in this region showed an overrepresentation of the oxygen transport process (e.g., oxygen binding and haptoglobin binding). We inferred and compared the localized haplotypes between DrFwB with both the counterpart (DrFW) and the bantam donor (DB). Within this introgressed region, the distribution of genotypes of ~ 2000 variants demonstrated that the sequence of DrFwB is highly homozygous and highly similar to the sequences from DB individuals (three out of six) (Fig. 4d).

## Discussion

### Complex and admixed population structure

In this study, we performed a set of genomic analyses in 37 Dutch chicken breeds of various management purposes for which whole-genome sequencing data has been generated. We observed that, in general, most Dutch native breeds were genetically closely-related to their bantam counterparts. However, it should be noted that the limited and imbalanced sample size between breeds may influence the interpretation of the results. Nevertheless, a close relationship was found between intermingled breeds, such as between breeds of the ornamental cluster (i.e., Brabanter (Brab) and Dutch Owl bearded (DOwBd)) and of the country fowl cluster (i.e., Frisian Fowl (FriFw), Assendelft Fowl (AssFw) and Groningen Mew (GrMw)). This pattern of close relationship between intermingled breeds has already been reported based on SNP-array data [3], confirming the substantial genetic similarity and potential gene flow between these breeds within clusters. One explanation for this observation is the similar selection for management types and shared morphological traits within clusters. By considering the history and features of the breed, we anticipated that the relatedness may be derived from the shared characteristics of these breeds, such as the shared form of the crest [17] or in case of the bantams within the clusters from the common bantam source. This relatedness is less likely to be associated with a geographic factor since Kraienkoppe Fowl (BreFw) and the breeds from Cluster 2 are not as geographically close as other breeds. We acknowledge that bantamization is one of the most important features in Dutch chicken breeds. In our previous bantam GWAS [11], we identified three bantam clusters with heterogeneous ancestral sources tracing back to: (1) Dutch bantam; (2) Sebright and Java bantam; and (3) bantams mainly with a South East Asian background. Interestingly, when comparing the two clustering approaches used in different studies, namely based on bantam ancestries and management histories, there was a general consensus on the subgroups to which individuals were assigned. We showed that although the two clustering approaches are based on different determinants, the assignments of breeds were still significantly correlated between the two clustering methods (see Additional file 2: Fig. S5, P < 2.2 × 10^–16^, Spearman’s correlation coefficient = 0.78). This observation suggested that the general management of Dutch breeds was partially confounded by the selective breeding for the bantam phenotype. This consensus highlighted the complexity of the population structure made up by multiple factors and the underlying relatedness between approaches. Although the applied clustering approaches should complement the objectives of our study, as we highlight here, it is important to take population substructure into account.

Taken together, the population structure of Dutch traditional chickens suggested a clear admixed substructure showing a complex ancestry. The influence of breed management on the substructure is pronounced. The genetic relationship between the three historical clusters may reflect the selection for breed standards and management categories which can be a major force subdividing the population structure and reshaping the characteristics of breeds during breed formation. Unlike other animals with more refined and specialized purebreds and well documented pedigrees (e.g., dogs), the local traditional chicken breeds are expected to have diverse genetic variations within populations. Furthermore, in the past, gene flow was extensive due to crossing with new breeds and later on gene flow occurred between breeds among local regions particularly with similar characteristics [17, 43].

### Management “barrier” between clusters

The results of isolation-by-distance demonstrated that genetic distance is correlated with geographic distance only within the clusters, which is likely due to gene flow being restricted within each cluster and to the management “barrier” between clusters. Given the limited dispersal distance between chickens in the Netherlands (maximum 227 km), strong artificial selective breeding may have created a major human-driven “barrier” that reshaped the genetic landscape over geographic distance. Nevertheless, when we look at dispersal distance within each cluster, breeds with a shared common management purpose can also be distributed across the country at a maximum distance of 200 km (with the exception of CL1 at a maximum dispersal distance of 80 km). When compared across clusters, management purposes differed and were unique, thus for breeds from different clusters at a close geographic distance, gene flow between them will be limited resulting in greater genetic distance. Consequently, the exclusive exchange of genetic materials within subdivided management clusters leads to a “barrier” between clusters. The limited haplotype sharing between clusters further confirmed the “barrier” between management groups which is likely due to restricted recent gene flow. Moreover, the haplotype sharing within clusters demonstrated some recent common ancestry and genetic exchange within clusters, rather than a deep phylogenetic split with restricted gene flow.

Overall, we observed a stronger effect of management than of geographic distance on the subdivision of the Dutch traditional chicken breeds. In other words, the breeds in the same management cluster tended to show a higher level of genetic similarity, outcompeting and masking the geographic separation at the whole population level. During the last century, selective breeding to maintain the characteristics of the traditional breeds has been strengthened in The Netherlands [3, 17]. Based on the historical management purposes of the breeds, these breed characteristics can be complicated because of the dynamic and changing breeding goal. One example is the coexistence of maintaining management types while breeding for the bantam phenotype. These diverse purposes are sometimes confounded or competing with each other, which complicates the overall genetic layout. Future studies that aim at disentangling the underlying interaction of factors involved in the population structure (e.g., between clusters CL2 and CL3) are needed. Moreover, it is common practice to increase phenotypic variation (e.g., color varieties) in the breeding goal for local traditional chicken breeds. It is important to note that the number of individuals from each breed in this study was relatively small, and more widespread sampling across the country, including more differentiated varieties, is necessary to confirm the results.

Although the productive specializations of breeds are generally homogenous, e.g., for egg production in CL1, it should be noted that breeds could have sub-divergent purposes, e.g., North Holland Blue was kept for its dual purpose i.e. egg-laying and production of meat [19]. The divergence within this cluster may subsequently have contributed to the genetic differentiation, therefore confounding with geographical distance.

### Detection of genomic regions with differentiation between breeds

We performed a genomic scan of FLK and hapFLK, which revealed the genomic regions selected for bantam and morphological features. Bantam phenotype is one of the known features under selection in Dutch populations and the identified signatures of selection were well supported by the body size variation. Both feather color and comb shape are phenotypes that are directly related with breed standards, and thus they are especially important for chickens kept for ornamental purposes and fancy breeding. According to previous studies and the OMIA (https://omia.org/home/) database for chicken, a deletion upstream of the *SOX10* gene is associated with dark/yellow brown feather color in chickens [44, 45], while an amplification of a duplicated sequence in the *SOX5* gene is responsible for the shape of the comb through the epistatic interaction with the *MNR2* gene [12]. The signatures of selection at these two genes suggested morphological diversification among Dutch breeds, reflecting the breeding interest associated with phenotypic variation. We further confirmed the known variants in the *SOX5* and *SOX10* genes. We confirmed the copy number variant (CNV) in the first exon of the *SOX5* gene in pea comb individuals and also demonstrated the deletion upstream of the *SOX10* gene in gold/yellow feather individuals and its absence in other plumage colors (see Additional file 2: Fig. S6). We noted that comb shape and plumage color are very variable within the Dutch breeds. For instance, there are over 20 different feather plumage varieties officially recorded in Dutch Bantam [19]. However, due to the limited sample size and incomplete phenotypic records, we were unable to validate all these signatures of selection or to refine the breakpoint of CNV. It is further important to note that the understanding of the signatures of selection (also from the transcriptomic level) is based on previous knowledge from the online catalogue OMIA. Improvement of the annotation of the phenotype database (e.g., through Functional Annotation of Animal Genomes (FAANG) [46]) will help the interpretation of signatures of selection.

### Impact of recent crossbreeding and “hitchhiking” introgressed segments

In many studies of introgression from an evolutionary perspective, the focus has been on the introgression event that happened many generations ago, often spanning thousands of years [10, 28, 47]. The three breeds used in crossbreeding (i.e., the donor breeds Drenthe Fowl and Dutch Bantam, and the derived breed Drenthe Fowl Bantam) provided us with an excellent proxy to investigate the recent event of hybridization which leaves many “hitchhiking” segments derived from the donor in the recipient genome. Based on our previous bantam GWAS [11], we identified *HMGA2* to be a candidate gene associated with the bantam phenotype and concluded that bantam within the Dutch population is a polygenetic trait with heterogeneous genetic backgrounds. Gene flow from bantam donors to derived neo-bantams was revealed around the associated *HMGA2* locus by haplotype sharing that has influenced the genomic makeup of bantam chickens. Here, we validated this bantam-associated region with a similar approach showing that relatively more haplotype segments were shared between the bantam donors and bantam recipient (neo-bantam) around *HMGA2* (Fig. 4b) than with normal-sized counterparts. In addition, we investigated the recent crossbreeding and the effect of “hitchhiking” introgression. An example is on chromosome 1 between 49.2 and 51.3 Mb (Fig. 4d), where haplotypes were observed to be diverse in DB, suggesting that the corresponding region is not completely fixed among individuals. In particular, the haplotype of two individuals comprised extensive heterozygous signals, which is more similar to the haplotype of normal-sized DrFw. This regional haplotype diversity in DB individuals implies that there is no clear evidence supporting specific selection for this region. One possible explanation for the consecutive introgressed signals in the genome of DrFwB is genetic drift. The DB introgressed haplotypes in DrFwB may be the result of genetic drift, which has subsequently been retained in the genome due to the short time since bantamization and/or the lack of recombination. We further compared this in another neo-bantam breed, Groningen Mew Bantam (GrMwB), which has a similar bantam crossbreeding origin (from DB) and has been established for a relatively long period (see Additional file 2: Fig. S7). Since positive selection and genetic drift are both expected to change the frequency of certain alleles or haplotypes, it should be noted that the observation of some of the fixed introgression signals may suggest potential selection or a coincided “hitchhiking effect”, which could be favorable for traits related to performance or morphology.

## Conclusions

Our findings provide insight in the complex population structure of Dutch chicken breeds and the factors that influence this complexity. The complex population structure can be attributed to selection for management standards and phenotypic differentiation, such as the crossbreeding processes applied to introduce the bantam trait. We also obtained evidence for the role played by geographic dispersal in the distribution of breeds within the subdivided populations. We detected a large set of signatures of selection that suggests diversifying selection in these breeds, including bantam-related selection and breed standard phenotypic selection. Finally, the DrFwB case study showed the power of genomic data to understand the recent demographic history and ongoing breed development.

## Supplementary Information

**Additional file 1: Table S1:** Dutch chicken breeds listed according to historical cluster and bantam groups. N is the number of animals sampled per breed.

**Additional file 2: Figure S1.** Unrooted Neighbor-Joining Tree (NJ-tree) illustrates the Reynold’s distance between breeds. **Figure S2.** Manhattan plot of hapFLK results. **Figure S3.** Distribution of shared identity-by-descent blocks identified between breeds. **Figure S4.** Distribution of regions of rIBD across the genome, showing the relative fraction of the haplotype sharing between DrFwB and DB and that between DrFwB and DrFw. **Figure S5.** Correlation of assignments of individuals based on the two clustering approaches. **Figure S6.** Demonstration of two CNV detected by signatures of selection in the *SOX5* and *SOX10* genes. **Figure S7.** Distribution of relative identity-by-descent (rIBD)on chromosome 1, showing the relative fraction of haplotype blocks shared between GrMwB and DB (positive rIBD) and GrMwB and GrMw (negative rIBD).

## Data Availability

Scripts used for relative identity-by-descent (rIBD) analyses are available on https://github.com/wzuhou/rIBD_WUR. Whole-genome sequence data were deposited in the European Nucleotide Archive (ENA). The data can be accessed through the two project numbers: (1) PRJEB34245, described 92 traditional chickens from the Netherlands in a previous study [48] and (2) PRJEB39725, the data of 44 Dutch chickens has been submitted [11].
