# Supplementary material for: Genomic insight into the influence of selection, crossbreeding, and geography on population structure in poultry
Source: Genet Sel Evol. 2023 Jan 20;55:5. doi: 10.1186/s12711-022-00775-x (PMC9854048; doi:10.1186/s12711-022-00775-x)
Supplement: Supplementary file 2 — Additional file 2: Figure S1. Unrooted Neighbor-Joining Tree (NJ-tree) illustrates the Reynold’s distance between breeds. Figure S2. Manhattan plot of hapFLK results. Figure S3. Distribution of shared identity-by-descent blocks identified between breeds. Figure S4. Distribution of regions of rIBD across the genome, showing the relative fraction of the haplotype sharing between DrFwB and DB and that between DrFwB and DrFw. Figure S5. Correlation of assignments of individuals based on the two clustering approaches. Figure S6. Demonstration of two CNV detected by signatures of selection in the SOX5 and SOX10 genes. Figure S7. Distribution of relative identity-by-descent (rIBD)on chromosome 1, showing the relative fraction of haplotype blocks shared between GrMwB and DB (positive rIBD) and GrMwB and GrMw (negative rIBD). [file 12711_2022_775_MOESM2_ESM.docx]

Additional Figures Wu et al

Page 2 Figure S1 Unrooted Neighbor-Joining Tree (NJ-tree) illustrates the Reynold’s distance between breeds.

Page 3 Figure S2 Manhattan plot of hapFLK results.

Page 4 Figure S3 Distribution of shared identity-by-descent blocks identified between breeds

Page 5 Figure S4 Distribution of regions of rIBD across the genome, showing the relative fraction of the haplotype sharing between DrFwB and DB and that between DrFwB and DrFw.

Page 6 Figure S5 Correlation of assignments of individuals based on the two clustering approaches.

Page 7 Figure S6 Demonstration of two CNVs detected by signatures of selection in SOX5 and SOX10.

Page 8 Figure S7 Distribution of relative identity-by-descent (rIBD)on chromosome 1, showing the relative fraction of haplotype blocks shared between GrMwB and DB (positive rIBD) and GrMwB and GrMw (negative rIBD).

Figure S1 Unrooted Neighbor-Joining Tree (NJ-tree) illustrates the Reynold’s distance between breeds. The colours of nodes on the phylogenetic tree show different breed groups, CL1 (yellow), CL 2 (green), CL3 (purple) and Lakenvelder (bantam) (red).

Figure S2 Manhattan plot of hapFLK results (A) across the genome. (B) Plot of hapFLK signals on chromosome 1 (GGA1), showing suggestive signatures of selection, highlighting genes HMGA2 (grey), SOX10 (yellow), SOX5 (red), as well as gga-mir-15a and gga-mir-16-1 (green).

Figure S3 Distribution of shared identity-by-descent blocks identified between breeds: between Dutch bantam and Drenthe fowl bantam (red) and between Drenthe fowl bantam and its normal-sized counterpart Drenthe fowl (blue).

Figure S4 Distribution of regions of rIBD across the genome, showing the relative fraction of the haplotype sharing between DrFwB and DB and that between DrFwB and DrFw.

Figure S5 Correlation of assignments of individuals based on the two clustering approaches.

The x-axis displays the three bantam clusters defined by bantam ancestries as described in Wu et al, [11]. In short, 1, Dutch bantam; 2, Sebright and Java bantam; and 3,bantams mainly with a South East Asian background. The y-axis shows the three clusters determined by the management types (as described in this study). The circles illustrate the assignment of breeds between two clustering approaches, with the size and colour of circles demonstrating the number of breeds for the assignment.


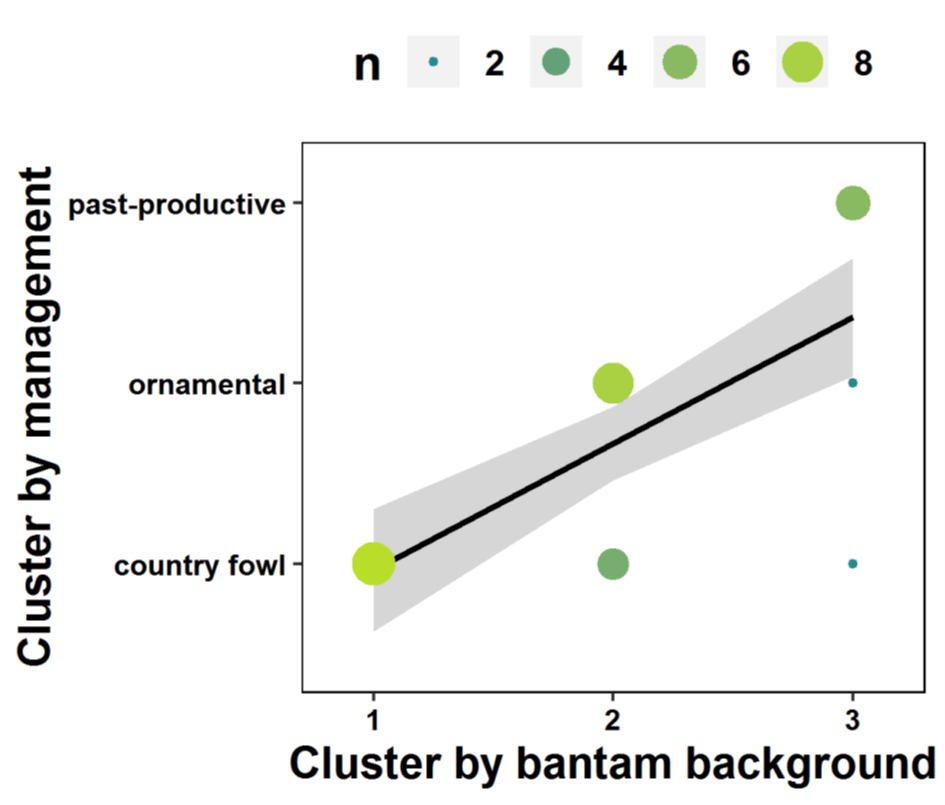


Figure S6 Demonstration of two CNVs detected by signatures of selection in SOX5 and SOX10.

(A) Coverage of sequence displays region on Chr1:65,811,401-65,973,900. Duplication in the first exon of SOX5 was found in the two pea comb chickens, whereas three single comb controls do not have it, the proximate interval is highlighted in yellow block(Chr1:65,837,500-65,846,000).

(B) Coverage of sequence displays region onChr1:51,030,958-51,065,558. Deletion located upstream of SOX10 was found in the two Gold feather chickens, whereas three controls with varied plumage colours do not have it, the proximate interval is highlighted in yellow block (Chr1:51,035,106-51,042,744)


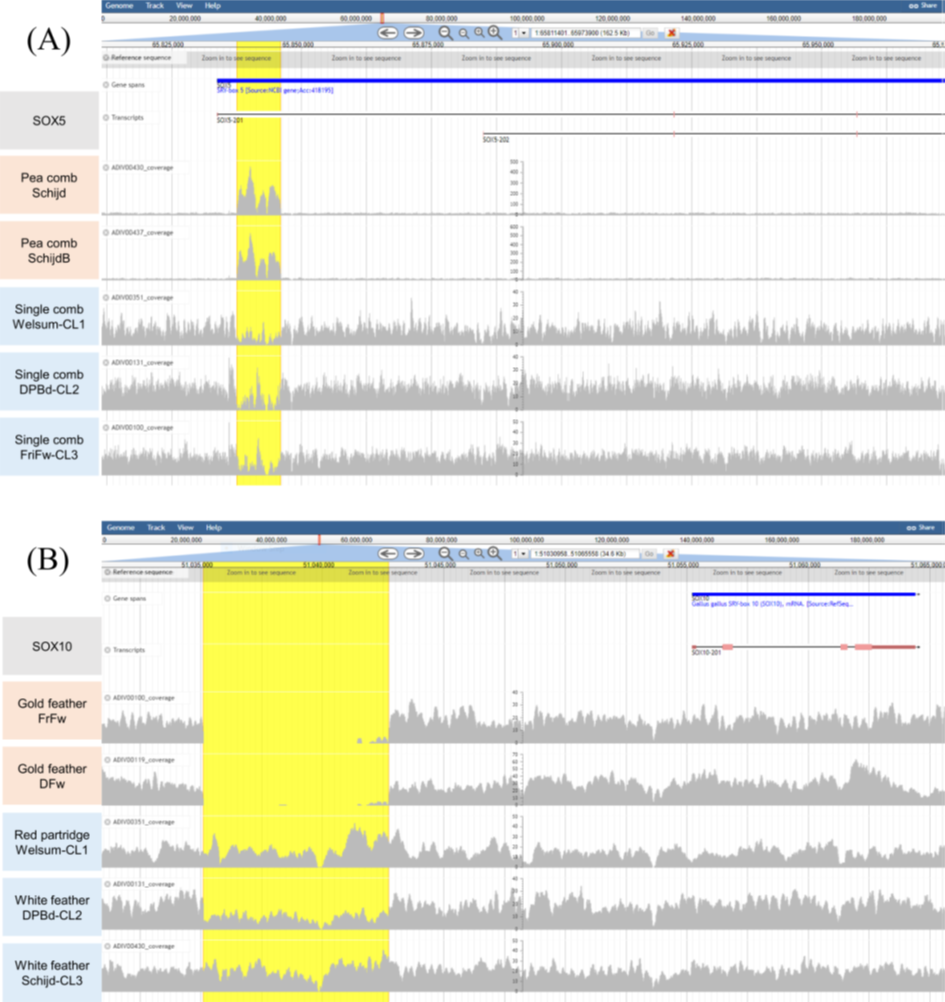


Figure S7 Distribution of relative identity-by-descent (rIBD)on chromosome 1, showing the relative fraction of haplotype blocks shared between GrMwB and DB (positive rIBD) and GrMwB and GrMw (negative rIBD). The HMGA2 related interval is highlighted by red dashed lines, the introgressed region (49.2-51.3Mb) is indicated by blue dashed lines.
